# Progress and Prospect of Zn Anode Modification in Aqueous Zinc-Ion Batteries: Experimental and Theoretical Aspects

**DOI:** 10.3390/molecules28062721

**Published:** 2023-03-17

**Authors:** Kaiyong Feng, Dongxu Wang, Yingjian Yu

**Affiliations:** College of Physics Science and Technology, Kunming University, Kunming 650214, China

**Keywords:** aqueous zinc-ion batteries, zinc dendrites, interface layer, zinc alloy, adsorption energy, molecular dynamics

## Abstract

Aqueous zinc-ion batteries (AZIBs), the favorite of next-generation energy storage devices, are popular among researchers owing to their environmental friendliness, low cost, and safety. However, AZIBs still face problems of low cathode capacity, fast attenuation, slow ion migration rate, and irregular dendrite growth on anodes. In recent years, many researchers have focused on Zn anode modification to restrain dendrite growth. This review introduces the energy storage mechanism and current challenges of AZIBs, and then some modifying strategies for zinc anodes are elucidated from the perspectives of experiments and theoretical calculations. From the experimental point of view, the modification strategy is mainly to construct a dense artificial interface layer or porous framework on the anode surface, with some research teams directly using zinc alloys as anodes. On the other hand, theoretical research is mainly based on adsorption energy, differential charge density, and molecular dynamics. Finally, this paper summarizes the research progress on AZIBs and puts forward some prospects.

## 1. Introduction

Limited energy resources have been a major problem facing the world over the past few decades. Currently, with rapid developments in science and technology, traditional fossil energy is on the verge of exhaustion; as a result, the demand for new energy sources, such as tidal, solar, wind, and nuclear power, has increased [1,2,3,4,5,6,7,8,9]. However, the direct application of these energy sources in daily life is difficult; thus, it is a better choice to convert them into electricity to power electronic devices [10,11,12]. Therefore, the development of energy storage devices with better performance is urgent and crucial. Compared with primary batteries, sustainable secondary batteries have attracted considerable attention in the battery industry. Among secondary batteries, lithium-ion batteries (LIBs) have been extensively used in portable electronics and electric vehicles owing to their long-term cycle and superior energy density [13,14]. Increases in the price of lithium resources have increased the cost of LIB production, thus limiting the application of LIBs in large-scale smart grids [15]. Zinc metal resources are more abundant in nature than lithium resources. Zinc is a highly reactive divalent metal in nature, and it possesses satisfactory specific capacity (820 mAh g^−1^) and a low electrochemical potential (−0.76 V compared with a hydrogen electrode standard). Metallic zinc has a hexagonal close-packed lattice structure, an ionic radius of 0.74 Å, and high electrical conductivity. In addition, Young’s modulus of metal zinc is 108 GPa, which is significantly larger than Na (10 GPa) and Li (5 GPa). The above value of 108 GPa shows that zinc exhibits smaller deformation when compressed or stretched; i.e., the zinc battery has better physical stability. Therefore, zinc metal batteries have better application prospects [16].

**Figure 1:**
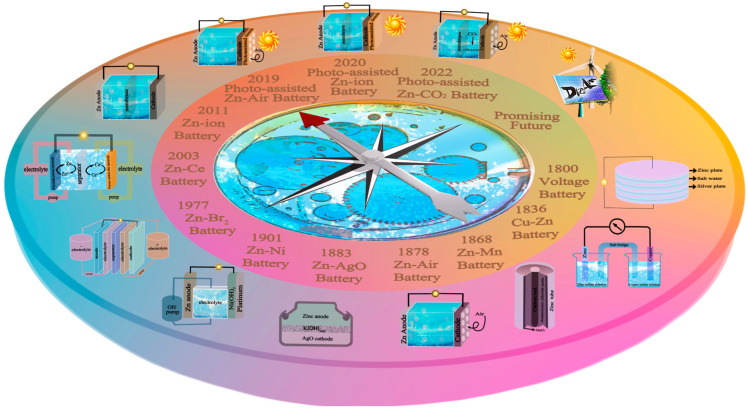
The development history of zinc-based batteries.

The history of the zinc-based battery dates back to 1800, when Alessandro Volta created the first zinc-based battery by stacking zinc and silver plates (Figure 1). Daniell invented the Zn-Cu battery (also known as the Daniell battery) in 1836. In 1868, Georges Leclanché made the first zinc-manganese wet battery using manganese dioxide and charcoal powder as a positive powder. The powder was pressed into a porous ceramic cylinder, and a charcoal rod collector was inserted as a positive electrode [17]. In 1878, researchers made the first air battery using a platinum-plated carbon electrode in place of the manganese dioxide electrode in the Leclanché battery. Clark’s 1883 patent reported the first completely alkaline silver-zinc oxide galvanic battery. In 1901, Thomas Alva Edison invented a rechargeable zinc-nickel battery system and obtained a U.S. patent. The zinc-bromine flow battery system was first developed and reported by H.S. Lim et al. in 1977. In 2003, Clarke proposed the zinc–cerium flow battery system. In 2011, Kang et al. first proposed the “zinc-ion batteries” (ZIBs) concept and reported reversible embed and exit of Zn^2+^ in MnO_2_. To date, ZIBs have gradually attracted the attention of researchers. In addition, the researchers have suggested that the use of light-assisted materials in batteries can directly introduce light into batteries and reduce energy loss in many intermediate conversion processes [18]. In 2018, Dai et al. reported the development of a photo-assisted zinc-air battery using a photo-responsive dual-function ORR/OER electrocatalyst [19]. In 2020, a photo-assisted ZIB that could directly charge via sunlight was proposed. The photo-assisted ZIB broadens the operating conditions and environment of zinc-ion batteries [20]. In 2022, photo-assisted zinc-CO_2_ batteries were proposed to mitigate the greenhouse effect [21].

In recent years, AZIBs have become popular owing to the advantages of zinc metal. AZIBs are mainly secondary batteries and an important class of aqueous batteries, with metallic zinc as an anode [22,23]. AZIBs are safer and cheaper to manufacture owing to their aqueous electrolytes. The electrolyte of the batteries does not pollute the environment, even when it is directly exposed to the air. Wang et al. proposed the assembly of safe AZIBs with broad application prospects using a drinkable solution of zinc gluconate as an electrolyte [24].

However, AZIBs have numerous defects. For example, the cathode of AZIBs is characterized by low capacity, severe capacity attenuation, and short service life [25,26]. At the same time, because of the critical role of the diaphragm in AZIBs, new problems arise, such as the slow migration rate of Zn^2+^ and the possibility of electrolyte freezing when AZIBs operate at low temperatures. The growth of dendrites close to the anode surface is the most severe problem associated with the zinc anode. In the process of charging and discharging, the inhomogeneous deposition of ions worsens the growth of dendrites [27,28,29]. Zinc dendrites also have a high Young’s modulus, which makes the membrane inside the battery easy to pierce, thus shortening the cycle life and decreasing the capacity of the battery. The anode surface passivation and corrosion greatly affect zinc deposition [30,31]. Considering the problems associated with AZIBs, many researchers have improved the performance of batteries by enhancing cathode capacity and zinc-ion migration rate and modifying the zinc anode. The modification of zinc anodes prevents the irregular growth of zinc dendrites, thus greatly enhancing the cycle life of the zinc anode. For example, various artificial interface layers have been constructed to guide the uniform distribution of Zn^2+^ on the anode surface. In addition, the artificial interface layer acts as a barrier between the anode and electrolyte, preventing the birth of side reactions [32,33,34,35]. Meanwhile, with the rapid development of computer technology, computational simulations have attracted considerable attention [36,37]. When the energy storage mechanism of batteries is investigated, theoretical calculations are used to analyze the electrochemical reaction processes from the atomic scale. The relationship between materials and performance can be elucidated effectively when theoretical calculations are combined with experimental techniques in research work. Theoretical calculations can also guide experiments in predicting electrode material properties and screening suitable electrodes, thereby saving time and eliminating trial-and-error costs [38].

**Figure 2:**
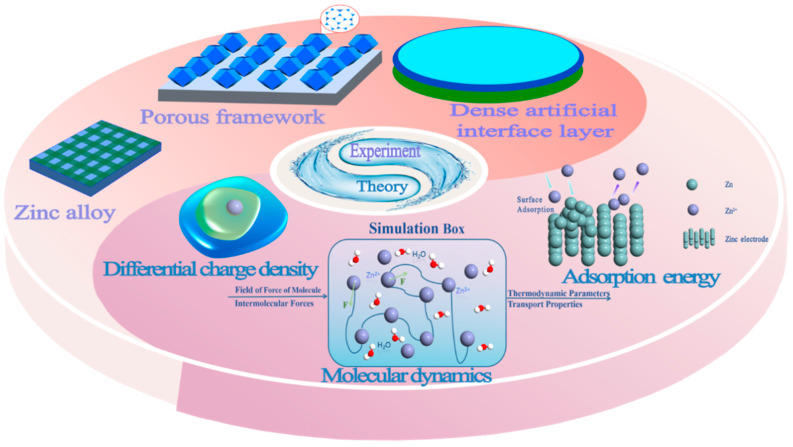
Experimental modification strategy and theoretical calculation method of a zinc anode.

Numerous excellent reviews of AZIBs have focused on the application of a specific class of materials in batteries, the energy storage mechanism, and aqueous electrolyte problems. However, few reviews have elucidated AZIBs from both experimental and theoretical aspects. Therefore, this review mainly summarizes the research of AZIBs from two aspects: experimental modification and theoretical calculation methods (Figure 2). The experimental modification methods are mainly divided into three categories: dense artificial interface layers, porous frameworks, and zinc alloys. These three kinds of experimental methods modify zinc anodes from different strategies. The theoretical research is mainly based on the interface adsorption energy, differential charge density, and molecular dynamics.

## 2. Basic Component and Energy Storage Mechanism

AZIBs comprise a cathode, an anode, an electrolyte, and a diaphragm. To help researchers to comprehensively understand how the components work, it is necessary to study the components of the battery. In addition, the investigation of the energy storage mechanism of batteries enables researchers to improve battery performance. In this section, the battery component, energy storage mechanism, and existing defects of AZIBs are briefly discussed from an electrochemistry perspective.

### 2.1. Basic Components of AZIBs

**Figure 3:**
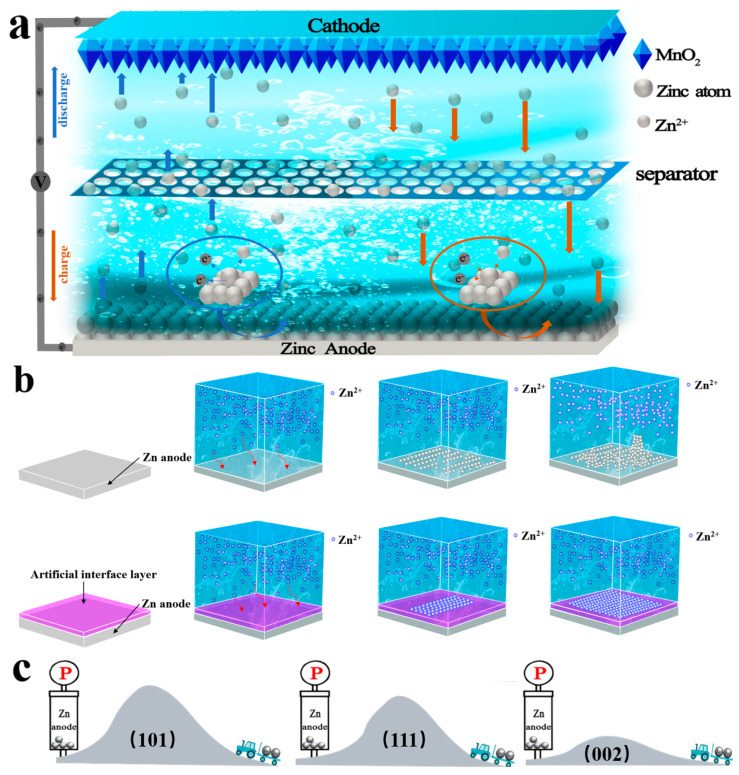
(**a**) Schematic diagram of charge and discharge processes of an aqueous zinc-ion battery. (**b**) Zinc dendrite growth process and inhibitory growth process after modification. (**c**) Diagram of the difficulty of zinc ion deposition in three crystal planes: (101), (111), and (002).

AZIBs are mainly composed of four parts: a negative electrode (anode), an electrolyte, a positive electrode (cathode), and a separator (Figure 3a). As the main body of Zn^2+^ deposition, the anode is generally obtained by processing zinc powder, foil, or flakes [39]. In contrast, the cathode has a more complex structure. The function of the cathode is to insert and extract Zn^2+^. It comprises active materials, conductive agents, and adhesives [40,41]. The electrolyte is a solution containing Zn^2+^, which is another source of Zn^2+^ in the battery. The battery separator prevents a short circuit resulting from the direct connection of the cathode and the anode, and it also provides a channel for Zn^2+^ to realize the intercommunication of Zn^2+^ between the anode and the cathode. AZIBs are also known as “rocking chair” batteries based on the movement path of Zn^2+^.

### 2.2. Energy Storage Mechanism of AZIBs

The energy of AZIBs is mainly provided by the movement of Zn^2+^. The charging and discharging principles are as follows (taking the manganese dioxide cathode as an example): when the battery discharges, the zinc metal on the anode surface releases electrons that enter the external circuit, generating Zn^2+^. Zn^2+^ ionized by the anode will enter the electrolyte and then increase the zinc-ion concentration at the anode terminal more than at the cathode terminal. The difference in zinc-ion concentration between the two electrodes promotes Zn^2+^ flux to pass through the diaphragm. In this process, H^+^ also passes through the diaphragm to balance out the difference in concentration between the two sides of the diaphragm. Therefore, the energy of AZIBs is also provided by H^+^ shuttling. At the same time, the cathode will acquire electrons from the external circuit and combine them with the Zn^2+^. In other words, Zn^2+^ in the electrolyte will be transported to the cathode to complete the discharge process (left half of Figure 3a). When the battery is charged, Zn^2+^ is released through the cathode into the electrolyte. At the same time, the cathode also releases electrons into the external circuit (right side of Figure 3a). The Zn^2+^ will pass through the separator again due to the difference in ion concentration. The Zn^2+^ close to the anode will combine with the electrons obtained from the external circuit and deposit on the anode surface [42]. Thus, the energy storage mechanism of AZIBs can be categorized into three parts: (1) the dissolution and deposition of Zn^2+^ on the anode surface, as described in Equation (1); (2) the insertion and extraction of Zn^2+^ at the cathode, as expressed in Equation (2); (3) the transport of Zn^2+^. The total working principle of AZIBs is expressed in Equation (3).

(1) $$\mathrm{Zn}↔{\mathrm{Zn}}^{2+}+{\;2e}^{-}$$

(2) $${\mathrm{Zn}}^{2+}+{2e}^{-}+{\;2\mathrm{MnO}}_{2}↔{\mathrm{ZnMn}}_{2}O_{4}$$

(3) $$\mathrm{Zn}+{2\mathrm{MnO}}_{2}↔{\mathrm{ZnMn}}_{2}O_{4}$$

### 2.3. Current Challenges of AZIBs

AZIBs have numerous natural advantages; however, there are still some challenges to be solved. The significant problem associated with the cathode is the poor capacity of materials. The capacity of the cathode material is evaluated by the storage capacity of Zn^2+^ in the cathode material and the decrease in storage capacity after multiple cycles. Compared with the cathode, the problems faced by the anode have more influence on the lifespan of AZIBs. The main problems associated with the anode are zinc dendrite formation and other side effects, such as corrosion and passivation. There are two kinds of side reactions in AZIBs. The first side reaction occurs in an alkaline electrolyte, as described in Equations (4) and (5). With the increase in pH value, the Zn(OH)_2_ precipitation in the electrolyte will decompose and produce ZnO. The final inert product (zinc oxide) is not conducive to Zn^2+^ migration. The other side reaction occurs in weakly acidic electrolytes, such as ZnSO_4_ solution, where hydrogen evolution reaction (HER) usually occurs, as described in Equation (6). The H_2_ produced via HER causes the battery to “expand.” Meanwhile, OH^−^ ion produced through HER can easily combine with SO_4_^2−^ and Zn^2+^ in the electrolyte to form another inert by-product, Zn_4_SO_4_(OH)_6_·nH_2_O, which is also unfavorable for zinc-ion transport, as described in Equation (7).

(4) $$\mathrm{Zn}+2{\mathrm{OH}}^{-}↔\mathrm{Zn}{\left(\mathrm{OH}\right)}_{2}+2e^{-}$$

(5) $$\mathrm{Zn}{\left(\mathrm{OH}\right)}_{2}↔\mathrm{ZnO}+{\;H}_{2}O$$

(6) $${2H}_{2}O+2e^{-}↔H_{2}+2{\mathrm{OH}}^{-}$$

(7) $${4\mathrm{Zn}}^{2+}+{\;\mathrm{SO}}_{4}^{2-}+6{\mathrm{OH}}^{-}+nH_{2}O↔{\mathrm{Zn}}_{4}{\mathrm{SO}}_{4}{\left(\mathrm{OH}\right)}_{6}\cdot nH_{2}O$$

The formation mechanism and inhibition strategies of zinc dendrites are attracting considerable scholarly attention. The Zn dendrite growth mainly occurs during the deposition of Zn^2+^, and the formation can be categorized into two steps: initial nucleation followed by subsequent growth [43,44]. The deposition of Zn depends on the interfacial Zn^2+^ concentration and nucleation site. The surface of a commercial Zn sheet is not completely flat, which indicates that the electric field distribution on the Zn matrix surface is not uniform when the zinc matrix is first deposited. Zn^2+^ is preferred to be deposited and forms small bumps in the higher potential of the zinc base. Then the process of zinc dendrite growth continues [45,46,47]. When the first zinc deposition is complete, the position potential of the initial convex spot will continue to increase because the “tip effect” makes the deposition of Zn^2+^ easier at the position. Finally, the “butterfly effect” intensifies to form dendritic deposition morphologies (zinc dendrites). Therefore, achieving uniform zinc-ion electrodeposition at the common Zn anode-electrolyte interface is difficult. During the cycling of AZIBs, the continuous and random growth of zinc dendrites perpendicular to the substrate eventually pierces the separator and causes the battery to short-circuit (Figure 3b). The migration rate of Zn^2+^ also affects the growth of dendrites. When the migration rate is low, the supply of Zn^2+^ close to the anode is limited during charging, which will also affect the uniform deposition of zinc.

Various modification strategies can be used to inhibit the Zn dendrite growth: first, the construction of an artificial interface layer or exposure of the 002-crystal surface of zinc metal to make the surface of the zinc anode equipotential [48]; second, the construction of a porous three-dimensional material on the anode surface to divide Zn^2+^ in the electrolyte and provide more reactive sites for uniform Zn^2+^ deposition. Another method is reducing Zn distribution on the anode surface and diverting Zn^2+^ in a disguised phase to inhibit the occurrence of zinc dendrites growth. For example, zinc alloy is used as the anode. Theoretical calculations on adsorption energy, differential charge density, and molecular dynamics are usually conducted to analyze the working principles of various modification strategies on Zn anodes. The experimental and theoretical studies on Zn anode modification are elucidated in the following sections [49,50,51].

## 3. Modification Strategy for Zinc Metal Anodes

At present, the research on zinc dendrites is still in its infancy. Various research groups have used different methods to modify zinc anodes to prevent the occurrence of zinc dendrite growth and the birth of side reactions. The modification methods for zinc anodes mainly include the construction of a dense artificial interface layer, the growth of a porous framework, and the preparation of zinc alloys. These strategies have been effectively used to modify the anode from different viewpoints to inhibit the growth of dendrites, with good results.

### 3.1. Dense Artificial Interface Layer

Dense artificial interface layers can be constructed using three strategies: (1) coating of the zinc substrate with an equipotential film. Thus, Zn^2+^ will be regularly deposited on the substrate surface. This type of film mainly mitigates the problem of surface electric field difference; (2) provision of more active sites for uniform zinc electrodeposition. The increased active sites can reduce the effect of a non-uniform electric field on zinc deposition on the anode surface. Furthermore, the deposition rates of Zn^2+^ on three crystal planes of zinc are different. At 0.02 eV Å^−2^, the 002-crystal surface energy of Zn is almost three times lower than the (111) and (101) surfaces (Figure 3c) [48,52]; (3) construction of artificial (002) crystal planes on the surface of zinc substrates to achieve uniform electrodeposition of zinc.

**Figure 4:**
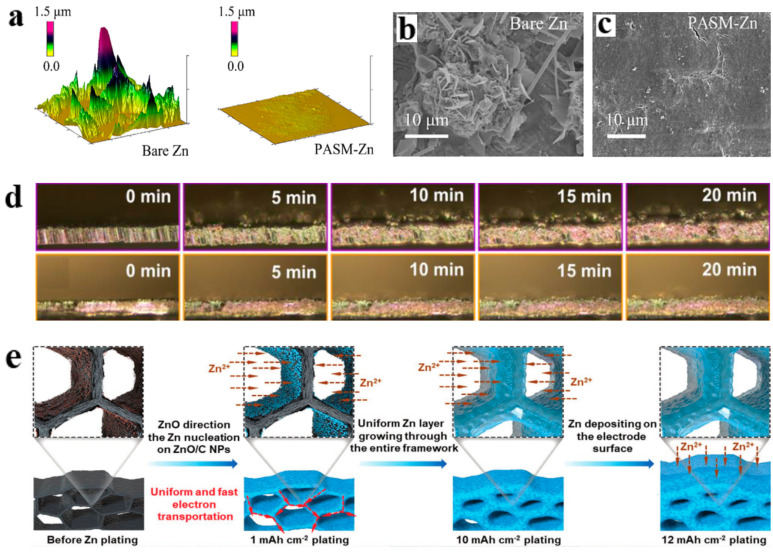
(**a**) AFM images and (**b**,**c**) SEM images of bare Zn and PASM-Zn after cycling at a current density of 1 mA cm^−2^ with an areal capacity of 1 mAh cm^−2^. Reproduced with permission [53]. Copyright 2022 Elsevier B.V. and Science Press. (**d**) In situ optical observation images of Zn deposition on the bare Zn and TCNQ@Zn electrode at 5 mA cm^−2^ for 20 min. Reproduced with permission [58]. Copyright 2022 Elsevier B.V. (**e**) Schematic illustration of Zn plating on the 3D-ZGC host in different states. Reproduced with permission [59]. Copyright 2022 Wiley-VCH GmbH.

Zn^2+^ electrodeposition on the anode surface is affected by many factors. For example, the electric field will affect Zn^2+^ because the Zn^2+^ is charged. After the equipotential film is deposited on the anode surface, the potential difference on the anode surface will be eliminated. Zn^2+^ is uniformly arranged on the anode surface induced by the uniform electric field. Yang’s group proposed a Ti_3_C_2_T_x_ MXene-assisted method of plating an equipotential film on the anode surface. With the assistance of MXene nanosheets, the anode surface was uniformly coated. The mechanical strength and ionic conductivity of the coatings were improved. The AFM image revealed that uniform electrodeposition of Zn^2+^ was realized after the surface of the Zn anode was coated (Figure 4a). A scanning electron microscopy (SEM) comparison diagram confirms that regular deposition of Zn^2+^ was achieved with the assistance of equipotential thin films (Figure 4b,c). The coated symmetric Zn anode battery had a long-term cycle of longer than 1900 h. Similarly, the full battery also exhibited good cycling steadiness of over 2600 cycles at 16 A g^−1^ [53]. He et al. coated the Zn surface with nano HfO_2_ to prevent the growth of dendrites. The nano HfO_2_ particles were evenly dispersed on the anode surface using polyvinylidene fluoride (PVDF). The electrochemical performance of the Zn@HfO_2_ was improved compared with that of the bare Zn. After detection, bare zinc had a contact angle of 93.2°, compared with a smaller contact angle of 78.1° for Zn@HfO_2_. The enhancement of hydrophilicity reduced the free energy of the Zn/electrolyte interface. In the Zn-MnO_2_ full battery test, the capacity of the bare zinc battery was only 37.9 mAh g^−1^. By comparison, the Zn@HfO_2_-MnO_2_ battery had a high capacity of 78.3 mAh g^−1^ [54]. In addition to the equipotential provided by the interface layers on the anode surface, another type of interface layer can provide numerous reactive sites. Hou et al. designed a dense artificial interface layer of high-yield carbon dots (CDs), which had abundant polar functional groups (CHO and C≡N) distributed on its surface. The interface layer provided many sites for accepting Zn^2+^ and guided Zn^2+^ to be evenly distributed on the anode surface. After the Zn@CDs composite anode was tested, its contact angle was only 63.8°. The layer of CDs had a strong affinity for Zn^2+^ and effectively induced uniform Zn^2+^ deposition. Moreover, the layer of CDs shielded active water and SO_4_^2−^ to prevent side reactions. At a current density of 1 mA cm^−1^, the life of zinc anodes can reach 3000 h [55]. Qian et al. fabricated Zn anodes with a dense InF_3_ coating (Zn@InF_3_). Compared with the contact angle between bare zinc and the electrolyte (64°), the contact angle between Zn@InF_3_ and the electrolyte reached 93.5°. The interface layer inhibited the side reaction of water on the anode surface. At the same time, high Zn^2+^ conductivity on the surface of the composite anode provided a perfect deposition environment. Electrochemical tests showed that the zinc anode could achieve over 6000 h of stable cycling at near 100% CE and the full battery with MnO_2_ as the cathode had a cycle life of up to 1000 times [56]. Choi et al. used artificial SEI formed by cross-linked gelatin to change the deposition morphology of zinc, which made the plating surface more uniform and denser. SEM analysis showed that the film was spatially chemically homogeneous and physically homogeneous. The contact angle of the coated zinc was much lower than that of the bare zinc (9.1° vs. 49°), indicating that the artificial SEI layer enhances the surface hydrophilicity. Meanwhile, the battery assembled with a gelatin-coated Zn anode had a lifespan of more than 250 cycles, compared with 63 cycles for the uncoated battery [57].

Electrodeposition of zinc was achieved on the (002) surface of polished single-crystal zinc (“Single-Zn”), which mainly utilized the defects of the crystal. Coating the surface with the lowest energy can solve the dendrite problem. In the study of using the (002) surface to modify the anode method, Robertson investigated limits of galvanizing single-Zn electrodes. The layered hexagonal planar Zn deposition was maintained when the plating conditions were increased from 2 mA cm^−2^ with an areal capacity of 1 mAh cm^−2^ to 10 mA cm^−2^ with an areal capacity of 8 mAh cm^−2^. Planar Zn deposition was still maintained even at 200 mA cm^−2^ [52]. Ye et al. prepared an anion (TCNQ^2−^) to modify the Zn anode. TCNQ^2−^ had the ability to continuously pump Zn^2+^ in the electrolyte owing to its numerous contained cyano groups. Meanwhile, the migration barrier of the (101) surface of the TCNQ^2−^-modified zinc anode was significantly higher than the (002) surface. Therefore, the TCNQ@Zn anode can restrain the deposition of Zn^2+^ on (101) and promote the diffusion deposition of Zn^2+^ along the (002) plane. Finally, a regular array perpendicular to the Zn (002) plane was formed on the anode surface. In other words, the modified anode favored the formation of ordered three-dimensional diffusion (002) plane deposition. The in situ growth of the bare zinc surface was not ideal, whereas the TCNQ@Zn electrode grew evenly under the same conditions (Figure 4d). At current densities of 1, 5, and 10 mA cm^−2^, the TCNQ@Zn anodes exhibited a life cycle of over 2000 h [58]. Zheng et al. introduced a dense interfacial layer of l-cysteine (Cys-Zn) with unique thiol groups. The dense interfacial layer improved the hydrophobicity of the zinc anode interface to guide Zn^2+^ uniform deposition and reduce the Zn corrosion reaction. Notably, l-cysteine etching of the zinc foil in situ preferentially exposed the (002) Zn plane and removed the native oxide layer on the Zn foil. Testing results showed that at a current density of 2 mA cm^−2^, the cycle life of the Cys-Zn@Zn anode-assembled symmetric battery was over 2000 h at 1 and 2 mAh [60].

### 3.2. Porous Framework

Researchers often construct a porous framework material on the Zn substrate, which enables Zn^2+^ to be separated and provides more active sites to deposit Zn^2+^ to prevent dendrite formation. For example, metal-organic frameworks (MOFs) have abundant porous structures and ultra-high specific surface areas. Therefore, MOFs and their derivatives can effectively control the migration, nucleation, and uniform deposition of Zn^2+^ when developing materials for modifying Zn anode surfaces [61]. Similarly, covalent organic frameworks (COFs) have the characteristics of large surface area, tunable porosity, high adsorption capacity, and porous structure, which have been applied to modify Zn anodes [62].

**Figure 5:**
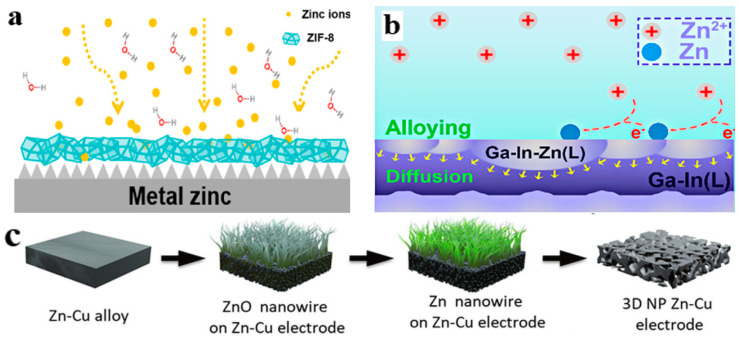

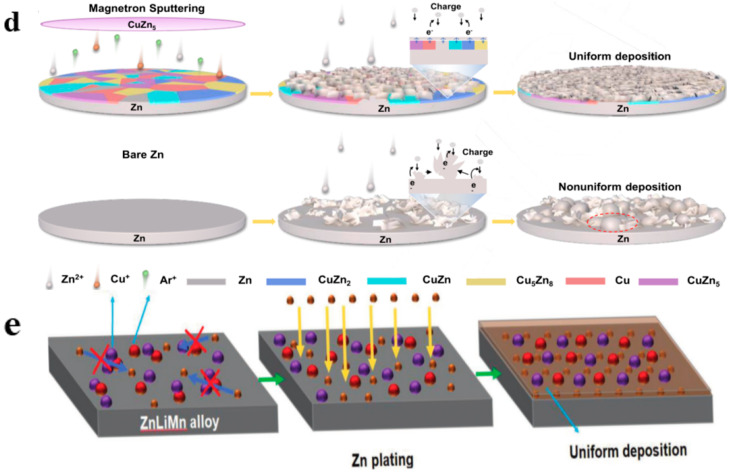
(**a**) Schematic diagram of zinc deposition on a Zn@ZIF-8 anode surface. Reproduced with permission [65]. Copyright 2021 ACS. (**b**) Dendrite-free GaIn@Zn anode by alloying-diffusion synergistic strategy. Reproduced with permission [67]. Copyright 2021 ACS. (**c**) 3D NP Zn-Cu alloy electrode fabrication process. Reproduced with permission [68]. Copyright 2020 WILEY-VCH Verlag GmbH & Co. KGaA, Weinheim. (**d**) Preparation process of Cu-Zn@Zn electrode, uniform Zn deposition on Cu-Zn@Zn electrode induced by Cu-Zn alloy layer, and nonuniform Zn deposition on bare Zn electrode owning to aggregation of metallic Zn. Reproduced with permission [69]. Copyright 2022 Wiley-VCH GDCH. (**e**) ZnLiMn alloy shows an even morphology during long-term stripping/plating process due to the electrostatic shield mechanism provided. Reproduced with permission [70]. Copyright 2022 Wiley-VCH GmbH.

Zhao et al. used a zinc-friendly COF to modify the anode. The stable zinc anode (TpPa-SO_3_H@Zn-foil) was obtained by preparing TpPa-SO_3_H films via interfacial reaction. The homogeneous deposition of Zn^2+^ was regulated by the coordination of -SO_3_H groups on the TpPa-SO_3_H film with Zn^2+^. Moreover, TpPa-SO_3_H could release a large amount of H^+^ and OH^−^ to reach a dynamic equilibrium, thus regulating the [Zn(H_2_O)_6_]^2+^ precipitation. In electrochemical tests, the CE of the composite electrode was approximately 99% after 1000 cycles. Symmetric anode tests were conducted at 1 mA cm^−2^ and 5 mAh cm^−2^, and the cycle life was over 1000 h, indicating excellent service life [63]. MOF-derived ZnO/C nanoparticles were decorated with 3D porous graphene-carbon nanotube scaffolds (3D-ZGC) with high electrical conductivity and uniform pores. 3D-ZGC is a typical synthesized porous framework material. The ultra-high specific surface area and large pore diameter provided sufficient storage space for zinc deposition and inhibited the uneven deposition of zinc (Figure 4e). At an ultra-high current density of 20 mA cm^−2^, the Zn@3D-ZGC electrode exhibited cycling steadiness of 1500 cycles with low overpotential (<65 mV) when tested in symmetric zinc anode batteries. In the full battery with a current density of 2000 mA g^−1^ (MnO_2_ as the cathode), the Zn@3D-ZGC//MnO_2_ battery maintained the capacity of 80.8% after 6000 cycles and the CE of 99.9% [59]. Wang et al. proposed the 3D silver-decorated zinc anode (Zn@Ag), achieving efficient and dendrite-free zinc plating. The alloying of the anode surface simultaneously suppressed HER and corrosion, improving the electrochemical stability of the silver mesh. The Zn/Ag meshes also exhibited good reversibility and cycling stability when they were tested with full batteries. Compared with the Zn//LFP battery, the Zn@ a-Ag mesh (Ag mesh electrode pretreated at 2 mA cm^−2^) //LFP (LiFePO_4_ cathode) battery had better performance in terms of charge and discharge capacity. Meanwhile, owing to the excellent electrical conductivity of silver, Zn^2+^ were uniformly attached to the a-Ag mesh surface during deposition. A complete battery composed of an a-Ag mesh-modified anode had long-term cycle stability [64]. As a type of zeolite imidazole framework (ZIF), ZIF-8, owing to a high porosity and specific surface area, is extremely stable in an aqueous solution and strong alkali solution. Therefore, Zn anode modified by ZIF-8 showed satisfactory performance. At 2 mA cm^−2^, the Zn@ZIF-8 symmetric battery can operate for over 1200 h. Furthermore, the Zn@ZIF-8 anode-based rechargeable Zn-ion battery exhibited ultra-long-term cycles without capacity decay over 10,000 cycles. As shown in the deposition diagram, Zn^2+^ was uniformly attached to the Zn@ZIF-8 surface with the assistance of the ZIF-8 material (Figure 5a) [65]. Hur et al. proposed a Nb_2_O_5_ film with uniform ion flux and fast transport channels to achieve uniform zinc electrodeposition. The material had a pore size similar to that of the MOF material, which regulates the homogeneous deposition of Zn^2+^. The composite anode (Nb_2_O_5_@Zn) exhibited extremely stable cycle life in the symmetrical anode system, i.e., plating/stripping up to 1000 h [66].

### 3.3. Construction of Zinc Alloy Anodes

Because of the strong activity of zinc metal, zinc is easily corroded in acidic or alkaline electrolytes. Alloying zinc metal with some corrosion-resistant metals can effectively inhibit corrosion. At the same time, the homogeneous distribution of zinc in the alloy system has a certain interval, which is also conducive to the homogeneous electrodeposition of zinc.

In a study of alloy anodes, Hou et al. constructed a multifunctional ZnSe protective layer (Zn@ZnSe). The ZnSe alloy evenly distributed Zn^2+^ and prevented the densification of zinc deposition. The surface of the alloy was composed of nanoparticles with uniform distribution. Therefore, the dense layered structure can inhibit the occurrence of side reactions. A symmetric battery assembled with Zn@ZnSe can achieve a cycle life of over 1500 h at 10 mA cm^−2^ with a capacity of 1 mAh [71]. Xue et al. used a zinc anode hybridized with a eutectic ZnAl alloy and Cu mesh (ZnAl@Cu-mesh). The Cu mesh was used as a support to provide a homogeneous electric field while guiding Zn^2+^ deposition. The electrochemical test revealed that the symmetric ZnAl@Cu-mesh//ZnAl@Cu-mesh battery exhibited well-behaved cycling stability (240 h at 0.5 mA cm^−2^) and low polarization (30 mV at 0.5 mA cm^−2^). Meanwhile, at 2 A g^−1^, the capacity retention rate of the ZnAl@Cu-mesh//V_2_O_5_ full battery was 95% after 2000 cycles. However, Zn//V_2_O_5_ failed after 750 cycles [72]. Chen et al. proposed a method of spontaneous replacement to generate a Cu_x_Zn_y_ alloy layer on the surface of a zinc anode. In the symmetrical battery test with ZnSO_4_ as the electrolyte, the anode cycle time was as high as 3800 h. The results showed that the alloy coating can guide the uniform deposition of zinc ions and effectively inhibit the growth of zinc dendrites. Meanwhile, the alloy zinc anode had a lower nucleation overpotential (32 mV) than that of bare zinc (93 mV) [73]. Ji et al. proposed a liquid gallium–indium alloy coating. The GaIn@Zn||GaIn@Zn battery had a cycle life of more than 2100 h at 0.25 mA cm^−2^ and 0.05 mAh cm^−2^. After the full battery (MnO_2_ as cathode) was put aside for 48 h, the battery had only 89.1% of the charging capacity; however, GaIn@Zn||MnO_2_ had 96% of the charging capacity. At the same time, GaIn@Zn||MnO_2_ had a good capacity preservation rate after countless cycles. The deposition process of Zn^2+^ at the GaIn@Zn anode indicated that the alloy surface combined with zinc metal to form a Ga-In Zn alloy (Figure 5b) [67]. Mai et al. fabricated a 3D nanoporous Zn-Cu alloy via electrochemically assisted thermal annealing (Figure 5c). First, the zinc in the Zn-Cu alloy became ZnO, which was subsequently removed, resulting in a Zn-Cu alloy electrode with numerous 3D pores. The structure of the electrode provided a wider ion transport path and more electrochemically active sites for uniform deposition/stripping of zinc. The high-voltage dual-electrolyte aqueous Zinc-Br_2_ battery prepared by them was close to typical commercial lithium-ion batteries. The maximum areal specific capacity of the battery was approximately 1.56 mAh cm^−2^ [68]. Kang et al. prepared Cu-Zn@Zn electrodes by taking advantage of high binding energy between Cu-Zn alloy layers and zinc atoms. The small contact area between the zinc metal and the electrolyte inhibited the aggregation of zinc atoms and promoted homogeneous deposition of Zn^2+^. The electrochemical tests conducted on the symmetric battery assembled with the Cu-Zn@Zn anode showed a long-term cycle of over 5000 h at 1mA cm^−2^ and 1mAh cm^−2^, and the capacity retention rate of the Cu-Zn@Zn//V_2_O_5_ full battery was 88% after 600 cycles. Uniform growth of Zn was achieved by depositing Zn^2+^ on the Cu-Zn@Zn alloy electrode (Figure 5e) [69]. Xie et al. used Li, Mn, and Zn to form an alloy. The shielding effect of Li and Mn prevented the transverse diffusion of Zn^2+^ from achieving uniform deposition. The ZnLiMn alloy exhibited uniform morphology during cycling (Figure 5d). The ZnLiMn||ZnLiMn battery had a cycle life of over 1000 h at 1 mA cm^−2^ and 1 mAh cm^−2^. In addition, the ZnLiMn||MnO_2_ full battery retained 96% of its initial capacity after 400 cycles at 1 C [70].

In studies of the anode of AZIBs, the electrolytes are generally ZnSO_4_ and Zn(OTf)_2_, according to the type of active material of the cathode [74]. Commonly used cathode materials include manganese-based materials and vanadium-based materials. AZIBs with manganese-based cathodes usually use ZnSO_4_ as an electrolyte, and a small amount of MnSO_4_ is added to improve the stability of the cathode during the whole battery test. Generally, Zn(OTf)_2_ is selected as the electrolyte for AZIBs composed of vanadium-based cathodes. The Zn(OTf)_2_ electrolyte can effectively inhibit the capacity attenuation of vanadium-based materials [75,76,77].

The performance of batteries assembled with a modified Zn anode is summarized in Table 1.

## 4. Theoretical Study on Modified Zinc Anode

The development of computer technology has made the application of theoretical calculation simulations more and more widespread. Density functional theory (DFT) is a classical method of studying the electronic structure of multi-electronic systems. It can be used in the judgment of structural stability, analysis of electronic structure, calculation of free energy of reactions, and so on. In the research of materials for energy storage, the relationship between structure and performance can be explained better by combining experimental characterization technology. Thus, researchers have applied DFT to calculate adsorption energy, charge density distribution, and molecular dynamics (MD) [104] to provide a detailed analysis of AZIBs at the atomic level [59,67,105].

### 4.1. Theoretical Study of Interfacial Adsorption Energy and Differential Charge Density

**Figure 6:**
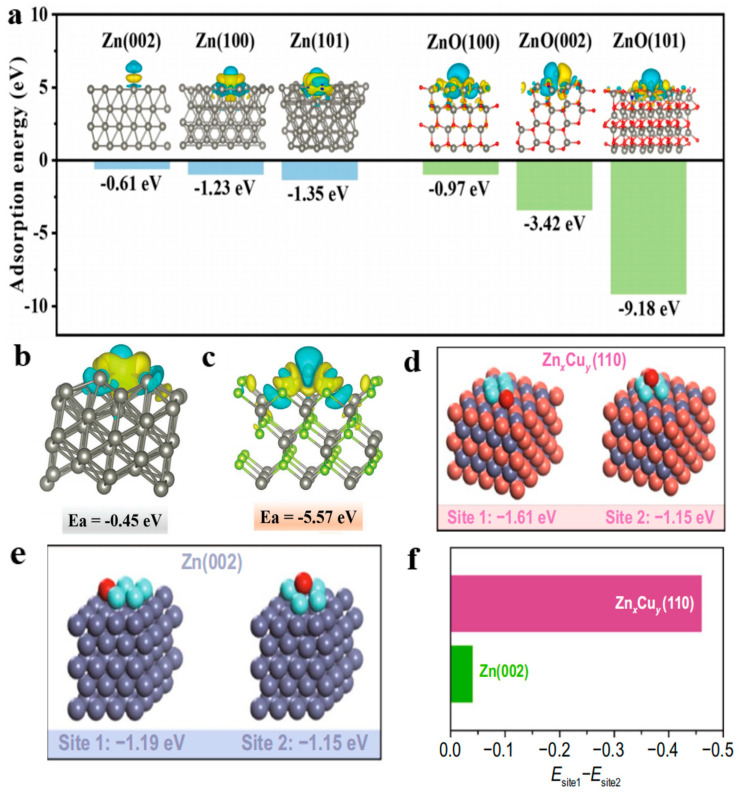
(**a**) DFT-calculated adsorption energies of Zn atoms on Zn and on ZnO crystal facets. Insets display the corresponding optimized atomic configurations of the adsorption complexes together with the differential charge density at the adsorption sites. Reproduced with permission [78]. Copyright 2022 Elsevier B.V. Differential charge density for the optimized structure of Zn^2+^ ion absorbed on (**b**) Zn (111) and (**c**) ZnSe (100) surfaces. The yellow and cyan regions represent charge accumulation and depletion, respectively. Reproduced with permission [79]. Copyright 2021 Wiley-VCH. Zn deposition at (**d**) side site (site 1) and (**e**) top site (site 2) on the Zn (002) and Zn_x_Cu_y_ (110) surfaces with different binding energies. (**f**) Comparison of energy difference between site 1 and site 2 at which Zn is deposited on the Zn (002) and Zn_x_Cu_y_ (110) surfaces. Reproduced with permission [80]. Copyright 2022 Springer.

**Figure 7:**
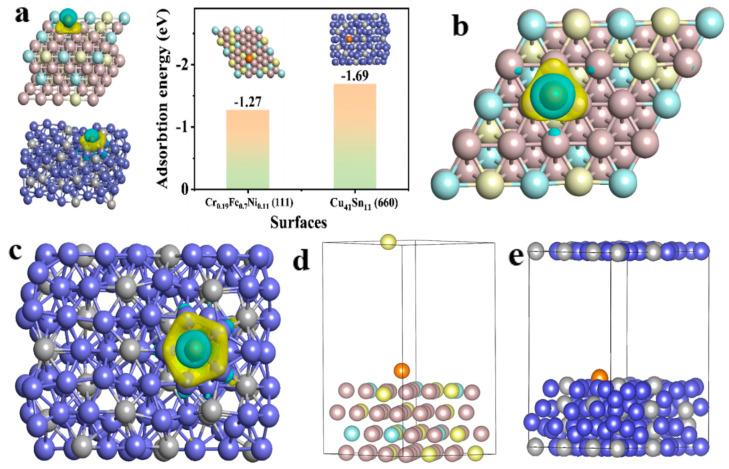
(**a**) The calculated differential charge density and adsorption energy of Zn^2+^, Cu_41_Sn_11_ (660), and Cr_0.19_Fe_0.7_Ni_0.11_ (111), in which yellow and cyan surfaces correspond to the charge gain and loss regions, respectively. The adsorption energy between the Zn atom and Cu_41_Sn_11_ (660) or Cr_0.19_Fe_0.7_Ni_0.11_ (111). The calculated differential charge density of Zn^2+^ on (**b**) Cr_0.19_Fe_0.7_Ni_0.11_ (111) and (**c**) Cu_41_Sn_11_ (660), in which yellow and cyan surfaces correspond to the charge gain and loss regions, respectively. The calculation models of Zn adsorbed on (**d**) Cr_0.19_Fe_0.7_Ni_0.11_ (111) and (**e**) Cu_41_Sn_11_(660). Reproduced with permission [81]. Copyright 2022 Elsevier B.V.

In the in-depth study of the electrode surface, adsorption energy calculation can be used to evaluate interface reaction efficiency. Researchers often analyze adsorption energy in cooperation with a potential diagram and charge density calculation, which can reflect the bonding properties and electronic coupling process. Hu et al. used nanosecond laser lithography to prepare zinc foils with periodic concave and convex patterns, which inhibited the growth of Zn dendrites [78]. The adsorption relationship between interfacial oxides and Zn^2+^ was further analyzed by DFT calculation (Figure 6a); low-index (002), (101), and (100) crystal planes of hexagonal zinc and zinc oxide structures were used to calculate the Zn^2+^ adsorption. The inset in Figure 6a shows the atomic structures of optimized adsorption complexes and the differential charge density (DCD) of the binding sites. The adsorption energies of the Zn atom on the low-index zinc oxide planes were significantly smaller than those on Zn planes. Adsorption energies of zinc atoms on the ZnO (101) and (002) planes were −9.18 and −3.42 eV, respectively, which were approximately 7 and 6 times the corresponding values on zinc planes. The adsorption energy of ZnO (100) was −0.97 eV, which was slightly higher than that of Zn (100). These results suggested that the ZnO surface was more Zn-friendly and exhibited stronger binding energy on the (101) and (002) ZnO planes. The chemical state of a zinc-friendly surface can facilitate dense nucleation and planar growth during initial deposition. Yang et al. reported a method of modifying zinc anodes using a simple selenization process, and the stable and dense zinc selenide layer was constructed. Because of the good zincophilic ability and natural ion diffusing channel of ZnSe, ZnSe@Zn achieved fast ion movement and uniform distribution of nucleation sites [79]. To reveal the interfacial ion diffusion process of the zinc selenide layer at the atomic level, the adsorption energies of Zn^2+^ cations on different zinc selenide and Zn planes were calculated. They constructed two representative interfacial adsorption geometries of ZnSe (100) and Zn (111) for comparison. The differential charge densities indicate that both substrates chemically interact with divalent Zn^2+^ via Zn-Se or Zn-Zn bonds (Figure 6b,c). The calculated adsorption energy of Zn^2+^ on the ZnSe (100) surface was −5.57 eV, which was smaller than that on the Zn (111) surface (−0.45 eV). The results show that ZnSe has better zincophilic ability than the bare Zn. Jiang et al. demonstrated that in situ alloying of surface Cu-Zn with the assistance of an anionic surfactant significantly improved the reversibility of 3D porous zinc electrodes [80]. The Zn_x_Cu_y_ alloy shell layer with zincophilic properties can guide the uniform deposition of Zn without nucleation overpotential and promote zinc stripping using Zn_x_Cu_y_/Zn electric coupling pairs. The Zn_x_Cu_y_ alloy shell layer endowed the nanoporous Zn_x_Cu_y_/Zn electrode with stable Zn stripping/plating behavior under aqueous electrolyte conditions. In addition, the zinc atoms were thermodynamically and uniformly deposited and parallel to the surface of the Zn_x_Cu_y_ alloy, which effectively inhibited the structure of zinc dendrites. DFT calculations showed that the Zn_x_Cu_y_ (110) surface had a special deposition location (site 1) in the early stage, whose adsorption energy was about −1.61 eV, which was ~0.46 eV lower than that of the top site (site 2) (~−1.15 eV) (Figure 6d–f). Meanwhile, Zn (002) had an energy deviation of ~0.04 eV at the site 1 and site 2 zinc deposition locations, indicating that the Zn_x_Cu_y_ alloy surface had more favorable-level growth than the monometallic Zn (002) plane (Figure 6f). Chen et al. designed a high-quality composite zinc mesh to fuel ultra-stable zinc batteries. A Cu–Sn alloy layer with a low nucleation barrier was first constructed (Cu-Sn@SSM) using a co-electrodeposition strategy to prevent dendrite formation [81]. Cu-Sn alloys were easily compounded with zinc and transformed into Zn-Cu alloys and Sn metals, which facilitated a more uniform ion distribution and a denser zinc deposition. Zn^2+^ was distributed on the surface of Cu_41_Sn_11_ (660) and Cr_0.19_Fe_0.7_Ni_0.11_ (111) (the main component of SSM), where the cyan and yellow surfaces correspond to the charge loss and gain regions, respectively (Figure 7a–c). The charge transfer from the surface of Cu_41_Sn_11_ to Zn^2+^ was more pronounced, which implies a stronger bond between the Zn ions and the surface of Cu_41_Sn_11_ (660). In addition, the adsorption energy corresponding to zinc atoms on Cu_41_Sn_11_ (660) was −1.69 eV, which is far less than that of Cr_0.19_Fe_0.7_Ni_0.11_ (111) surface (−1.27 eV) (Figure 7a). Figure 7d,e show the calculated models for Zn adsorption on both surfaces. These results showed that zinc was preferentially plated on the Sn-Cu layer rather than the original steel mesh, which provided a theoretical basis for the experiments. Thus, the calculation of adsorption energy can be used to study the strength of ligand interaction with the surface. It can also be used to evaluate the selectivity of ligands on the active site and different crystal planes. The calculation of differential charge density can further verify or predict the adsorption process on the anode surface.

### 4.2. Molecular Dynamics

**Figure 8:**
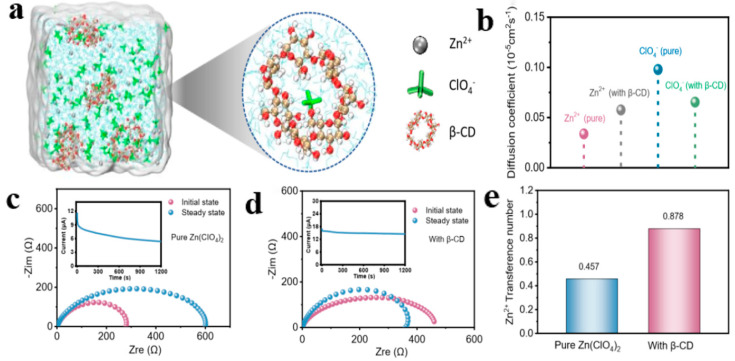
Theoretical simulations and electrochemical tests for explaining fast Zn^2+^ diffusion modulated via *β*-CD@ ClO_4_^−^ complex. (**a**) 3D snapshot of Zn(ClO_4_)_2_ system with the *β*-CD additive gathered from MD simulations and partial enlarged snapshot representing ClO_4_^−^ anion bind with *β*-CD successfully; (**b**) Diffusion coefficient of Zn^2+^ and ClO_4_^−^ in two above electrolyte systems (with and without *β*-CD additive) derived from MD simulations. EIS of the Zn||Zn symmetric batteries (**c**) without and (**d**) with the *β*-CD additive before and after polarization; inset: variation of current with time during polarization at an applied voltage of 10 mV at room temperature.(**e**) Zn^2+^ transference number (tZn^2+^) comparison of two above electrolyte systems. Reproduced with permission [109]. Copyright 2022 Wiley-VCH.

**Figure 9:**
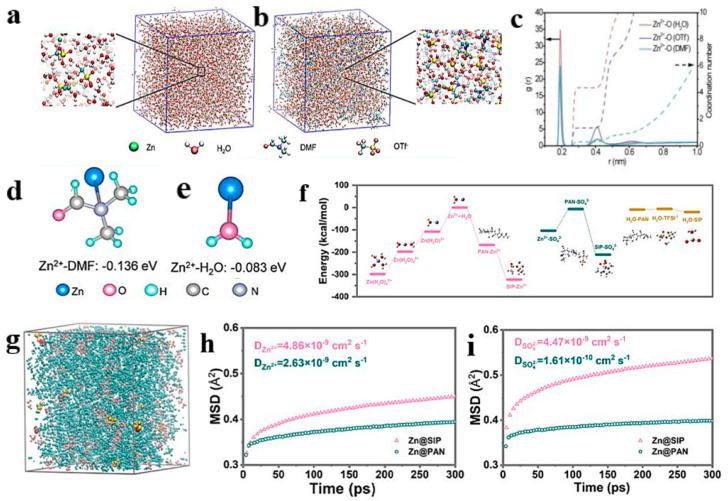
Snapshot of the MD simulation batteries for (**a**) ZOTF and (**b**) ZOTF-2H1D electrolytes. (**c**) RDFs of Zn^2+^-O (H_2_O), Zn^2+^-O (OTf^−^), and Zn^2+^-O (DMF) pairs, and the coordination number in the ZOTF-2H1D electrolyte. The calculated adsorption energy of Zn^2+^ with (**d**) DMF molecule and (**e**) H_2_O molecule. Reproduced with permission [110]. Copyright 2022 Wiley-VCH. Desolvation process of hydrated Zn^2+^ and ion transport mechanism in the SIP polymer interface. (**f**) Calculated bonding energy in the desolvation and transport processes of Zn^2+^. (**g**) Snapshot of ion transport through SIP coating interface via MD simulation. (**h**,**i**) Simulated mean square displacement of Zn^2+^ and SO_4_^2−^ in SIP and interface layer coupling polyacrylonitrile (PAN) skeleton coating. Reproduced with permission [111]. Copyright 2022 Wiley-VCH.

AZIBs form dendrites on the Zn electrode during charge and discharge resulting from uncontrollable nucleation and slow Zn^2+^ kinetics at the negative zinc interface. MD simulation has become a reliable tool for studying molecular and atomic systems under specific environmental conditions in computational work. From the perspective of dynamics, the evolutionary behavior of the system is studied by simulating the motion states of atoms and molecules within a certain time [106,107,108]. Inspired by host-guest interactions, Zhi et al. reported a strategy for anion trapping in AZIBs. The anion trap β-cyclodextrin (β-CD) was added into the Zn(ClO_4_)_2_ electrolyte for inducing Zn (002) deposition and promoting the migration behavior of Zn^2+^ [109]. MD simulations were performed to explore the effect of the interaction between the anion and *β*-CD on the cation and anion transport kinetics (Figure 8a,b). In the pristine electrolyte, the diffusion rate of Zn^2+^ was lower than that of ClO_4_^−^ because the six water molecules of Zn^2+^ were strongly solventized, and the coupling between the cation and anion pairs further hindered the transport of cations. In addition, the inner cavity of *β*-CD spatially restricted the migration of ClO_4_^−^ anions, leading to the uncoupling of Zn^2+^-ClO_4_^−^ ion pairs, thus transforming the *β*-CD-modified electrolyte into a restricted state with high mobility (Figure 8c,d). In further investigation, the Zn^2+^ migration value of the Zn (ClO_4_)_2_-*β*-CD electrolyte was 0.878, which is much higher than the migration value of pure Zn (ClO_4_)_2_ (0.457) (Figure 8e). The increased zinc-ion mobility led to a stable and sufficient zinc flux between the natural electrolyte environment and the electrode-electrolyte interface, thus increasing reaction efficiency on both sides of the two electrodes. Starting from the classical chemical theory of Lewis acid–base theory, Lu et al. used unpaired electrons to rivet Zn^2+^ while breaking the intermolecular hydrogen bonds of free water molecules and reducing their reactivity to enhance the electrochemical performance of AZIBs. They performed MD simulations of the solventized structure of [Zn (H_2_O)_6−x_(OTf^−^)_x_]^2+^ with hydrated Zn^2+^ and OTf^−^ to accurately investigate the effect of N-dimethylformamide (DMF) additives on the solventized structure of Zn^2+^ (Figure 9a). DMF can be ligated with Zn^2+^ as part of the solventized structure (Figure 9b). In the ZOTF-2H1D electrolyte, the initial Zn^2+^ solventized sheath layer contains 4.4 H_2_O molecules, 1.3 OTf^−^ anions, and 0.3 DMF molecules, which can be expressed as [Zn (H_2_O)_4.4_(OTf^−^)_1.3_(DMF)_0.3_]^2+^. H_2_O molecules were removed from the Zn^+^ solvated sheath, thus inhibiting HER and Zn dendrite growth and electrode corrosion (Figure 9c) [110]. The researchers conducted the DFT calculation method to examine the effect of DMF addition on the solventized sheath layer of Zn^2+^. The optimal coordination structure was formed by Zn^2+^ and water molecules (Figure 9d,e). The adsorption energy of DMF for Zn^2+^ was smaller than that of water (−0.136 eV vs. −0.083 eV). Due to the zincophilic ability nature of DMF, the addition of DMF changed the solventized structure of Zn^2+^ and promoted the binding of Zn^2+^ to DMF and OTf^−^ ion. Chen, Qiu, and Hou fabricated a semi-immobilized ionic liquid interface (SIP) for achieving thermodynamic stabilization of zinc anodes and rapid Zn^2+^ transport over a large temperature ranging from −35 to 60 °C [111]. To understand the desolvation process and the ion transfer on the SIP interface, the bonding energies of the different components were determined using DFT calculations and theoretical conformational models (Figure 9f,g). The results showed that the shell layer of H_2_O molecules in [Zn(H_2_O)_x_]^2+^ solvation was blocked, and the Zn^2+^ cluster was transformed into the SIP–Zn structure. The Zn^2+^ transport in the SIP layer was further studied using MD simulations (Figure 9h,i). The results revealed that Zn^2+^ and SO_4_^2−^ exhibited high diffusion coefficients in the SIP layer, suggesting that the SIP layer facilitated Zn^2+^ and SO_4_^2−^ ion diffusing and improved the ion transfer kinetics. Excellent interfacial compatibility and fast transfer kinetics are key issues for zinc electrode and SIP interfaces, which can be promoted by enhancing ion desolvation and accelerating ion transfer. In conclusion, through the above application cases, MD simulation is a very promising prospect in the exploration of electrolyte structure, ionic conductivity, and other physical and chemical properties. It can also be used to study interfacial reaction mechanism.

## 5. Summary and Outlook

**Figure 10:**
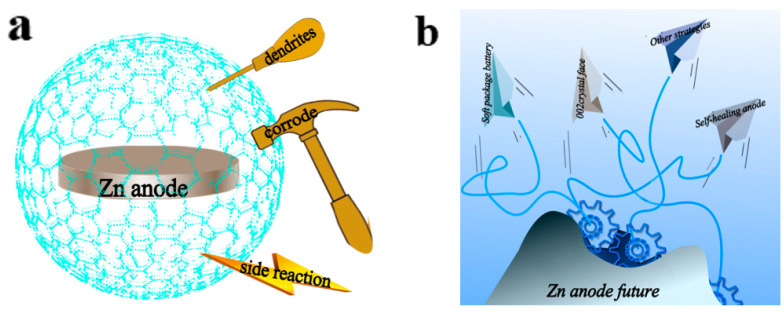
(**a**) Schematic of anode protection. (**b**) Diagram of the possible development directions of zinc anodes in the future.

In this review, we first introduced the basic challenges associated with anodes used in assembling AZIBs and then discussed various modification strategies for the anodes in detail. Specifically, problems such as uncontrollable growth of Zn dendrites, corrosion, and water-side reaction significantly affect the battery life cycle. Then experimental construction methods of the Zn metal anode interface layer and research progress on anode surface modification were discussed in terms of three aspects: dense artificial interface layers, porous frameworks, and zinc alloys. Various materials, including carbon, polymer, metal oxides, metals and alloys, and MOF materials, have been used to modify the Zn anode. In detail, the Zn anode can be protected by (i) preventing direct contact between the anode and the electrolyte; (ii) preventing the occurrence of water-side reactions; (iii) balancing anode surface potential; (iv) establishing modulated ion channels; (v) increasing contact area and providing more active sites; and (vi) constructing zinc alloy anodes. With the help of the modified material, the zinc anode obtains a protective cover to prolong its service life (Figure 10a).

The theoretical studies were mainly based on DFT, which only required atom types and positions. In the theoretical studies, scientists mainly calculated the interface adsorption energy, differential charge density, and MD. Theoretical studies help researchers to comprehensively understand the relevant experimental phenomena. In recent years, machine learning has been gradually applied to material discovery, property prediction, and other fields to enhance investigative efficiency and reduce the cost of the experiment. In the future, machine learning will play a significant role in exploring AZIBs [112].

Although several studies have been conducted on anode materials for AZIBs, development is still in its infancy. For example, when constructing an artificial interface layer, it is indispensable to consider the coating thickness, the interface bonding ability, the mechanical structure, and the service life. These issues still exist and need to be resolved. Thus, this review suggests possible future developments for Zn anodes (Figure 10b). Considering the material cost, structural complexity, and performance exhibited by the modified battery, the construction of an interface layer to reduce the migration barrier of the (002) face might be one of the possible commercialization routes. At the same time, a new strategy called self-healing was proposed. It is a promising and effective strategy for removing dendrites, inhibiting parasitic reactions, and improving cycling stability of Zn batteries. However, the reports on self-healing strategies are still limited. Compared with other modification strategies, the zinc anode with self-healing behavior can completely solve the problem of short service life. Therefore, the self-healing modification strategy has more commercial prospects in the face of the complex working environment and different job requirements. Flexible zinc-ion batteries that can change their appearance and shape will have a big market. However, the high mechanical strength of zinc metal makes it difficult to make soft-pack batteries. For such problems, the flexibility of zinc anodes can be improved by doping small amounts of other elements. The soft zinc anode allows higher plasticity in unconventional batteries. Moreover, the life cycle of zinc anodes at extreme temperatures should be considered. Due to the solidification of low-temperature electrolytes, the transport speed of zinc ions is limited. During charging, the low ion transport rate results in slight distribution of Zn^2+^ close to the anode surface. Thus, a small amount of Zn^2+^ close to the anode is randomly deposited to produce dendrites. Considering the dendrite growth with extremely low Zn^2+^ content, a kind of interfacial layer can be studied to guide ion movement. Unlike the deposition of Zn^2+^ at high concentrations, the interface can uniformly grow on the anode surface even at extremely low ion concentrations.

We believe this review provides valuable information for modifying zinc anodes for AZIBs.

## Figures and Tables

**Table 1:** Performance of AZIBs with a modified zinc anode (2021–2023).

Modification Strategy			Performance					Refs.
		Full Battery		Half Battery				
	Electrolyte	Current Density	Capacity Retention Rate	Electrolyte	Area Specific Current	Area Specific Capacity	Time	
Ti_3_C_2_T_x_ MXene	ZnSO_4_ + Na_2_SO_4_	16 A g^−1^	85.0% after 2600 cycles	ZnSO_4_	0.20 mA cm^−2^	0.20 mAh cm^−2^	2000 h	[53]
Zn@CDs	ZnSO_4_	5 A g^−1^	81.6% after 500 cycles	ZnSO_4_	1.00 mA cm^−2^	1.00 mAh cm^−2^	3000 h	[55]
Zn@InF_3_	ZnSO_4_ + MnSO_4_	3C	80.0% after 1000 cycles	ZnSO_4_	0.50 mA cm^−2^	0.50 mAh cm^−2^	6000 h	[56]
TCNQ@Zn	\	\	\	ZnSO_4_	1.00 mA cm^−2^	1.00 mAh cm^−2^	2000 h	[58]
3D-ZGC@Zn	ZnSO_4_	2 A g^−1^	80.8% after 6000 cycles	ZnSO_4_	10.0 mA cm^−2^	1.00 mAh cm^−2^	400 h	[59]
Cys-Zn@Zn	ZnSO_4_	1 A g^−1^	78.7% after 1000 cycles	ZnSO_4_	2.00 mA cm^−2^	2.00 mAh cm^−2^	2000 h	[60]
TpPa-SO_3_H@Zn	ZnSO_4_	5 mA cm^−2^	94.7% after 1000 cycles	ZnSO_4_	1.00 mA cm^−2^	5.00 mAh cm^−2^	1000 h	[63]
Zn@ZIF-8	ZnSO_4_ + MnSO_4_	0.5 A g^−1^	76.0% after 250 cycles	ZnSO_4_	10.0 mA cm^−2^	1.00 mAh cm^−2^	5000 h	[65]
Nb_2_O_5_@Zn	Zn(OTf)_2_	2 A g^−1^	78.6% after 500 cycles	ZnSO_4_	1.00 mA cm^−2^	0.50 mAh cm^−2^	1000 h	[66]
GaIn@Zn	ZnSO_4_	\	\	ZnSO_4_	0.25 mA cm^−2^	0.05 mAh cm^−2^	2100 h	[67]
Cu-Zn@Zn	Zn(OTf)_2_	2 A g^−1^	88.2% after 600 cycles	Zn(OTf)_2_	1.00 mA cm^−2^	1.00 mAh cm^−2^	5496 h	[69]
ZnLiMn	ZnSO_4_ + MnSO_4_	1C	96.0% after 400 cycles	ZnSO_4_	1.00 mA cm^−2^	1.00 mAh cm^−2^	1000 h	[70]
Zn@ZnSe	\	\	\	ZnSO_4_	1.00 mA cm^−2^	1.00 mAh cm^−2^	1500 h	[71]
ZnAl@Cu-mesh	Zn(OTf)_2_	2 A g^−1^	95.0% after 2000 cycles	Zn(OTf)_2_	0.50 mA cm^−2^	0.25 mAh cm^−2^	240 h	[72]
Zn@Zn_x_Cu_y_	\	\	\	ZnSO_4_	0.25 mA cm^−2^	0.25 mAh cm^−2^	3800 h	[73]
LLP@ Zn-foil	ZnSO_4_ + MnSO_4_	1 A g^−1^	75.0% after 500 cycles	ZnSO_4_	0.50 mA cm^−2^	0.50 mAh cm^−2^	1100 h	[78]
ZnSe@Zn	\	\	\	ZnSO_4_	1.00 mA cm^−2^	0.50 mAh cm^−2^	1700 h	[79]
Zn_x_Cu_y_@Zn	Zn(OTf)_2_ + SDS + Mn(OTf)_2_	1 A g^−1^	84.0% after 800 cycles	Zn(OTf)_2_	0.50 mA cm^−2^	0.50 mAh cm^−2^	1900 h	[80]
Zn@Cu-Sn@SSM	Zn(OTf)_2_	2 A g^−1^	84.0% after 1000 cycles	ZnSO_4_	10.0 mA cm^−2^	3.00 mAh cm^−2^	1050 h	[81]
Zn-TCPP@Zn	Zn(OTf)_2_	4 A g^−1^	82.5% after 1000 cycles	ZnSO_4_	0.20 mA cm^−2^	0.20 mAh cm^−2^	2600 h	[82]
UiO-67-2D	ZnSO_4_	2 A g^−1^	81.0% after 1500 cycles	ZnSO_4_	3.00 mA cm^−2^	0.50 mAh cm^−2^	800 h	[83]
Zn@ZIF-L	Zn(OTf)_2_	0.5C	84.9% after 250 cycles	Zn(OTf)_2_	0.25 mA cm^−2^	0.25 mAh cm^−2^	800 h	[84]
CuZIF-L@TM/Zn	\	\	\	ZnSO_4_	1.00 mA cm^−2^	1.00 mAh cm^−2^	1100 h	[85]
Zn@TiO_2_/NC	ZnSO_4_	0.5 A g^−1^	75.0% after 1000 cycles	ZnSO_4_	5.00 mA cm^−2^	1.00 mAh cm^−2^	1100 h	[86]
Zn@ZnS/NC	\	\	\	ZnSO_4_	0.20 mA cm^−2^	0.50 mAh cm^−2^	2000 h	[87]
ZIF-8@Zn	ZnSO_4_	5.0 A g^−1^	96% after 13,000 cycles	ZnSO_4_	2.00 mA cm^−2^	2.00 mAh cm^−2^	800 h	[88]
Zn@CCF	\	\	\	ZnSO_4_	4.40 mA cm^−2^	4.40 mAh cm^−2^	1200 h	[89]
Zn@LM	ZnSO_4_ + MnSO_4_	1 A g^−1^	90.0% after 1000 cycles	ZnSO_4_	0.50 mA cm^−2^	0.50 mAh cm^−2^	800 h	[90]
Zn@ZnP-NC	\	\	\	ZnSO_4_	2.00 mA cm^−2^	1.00 mAh cm^−2^	1100 h	[91]
Silk II-SF@Zn	\	\	\	ZnSO_4_	10.0 mA cm^−2^	10.0 mAh cm^−2^	3300 h	[92]
ZnAl	Zn(OTf)_2_	5 A g^−1^	95.0% after 1000 cycles	Zn(OTf)_2_	0.50 mA cm^−2^	0.25 mAh cm^−2^	300 h	[93]
Zn@Cu-HHTP@MX	\	4 A g^−1^	92.5% after 1000 cycles	\	\	\	\	[94]
Zn@ZnF_2_	\	\	\	ZnSO_4_	1.00 mA cm^−2^	1.00 mAh cm^−2^	2500 h	[95]
NOC@Zn	\	\	\	ZnSO_4_	1.00 mA cm^−2^	1.00 mAh cm^−2^	3040 h	[96]
Zn@GDY	\	\	\	ZnSO_4_	10.0 mA cm^−2^	1.00 mAh cm^−2^	16,000 h	[97]
Zn@UiO-66-(COOH)_2_	ZnSO_4_	1 A g^−1^	91.0% after 2400 cycles	ZnSO_4_	2.00 mA cm^−2^	2.00 mAh cm^−2^	2800 h	[98]
PPA-Zn	ZnSO_4_ + MnSO_4_	1 A g^−1^	78.0% after 500 cycles	ZnSO_4_	2.00 mA cm^−2^	1.00 mAh cm^−2^	6500 h	[99]
*β*-PVDF/BMI@Zn	\	\	\	ZnSO_4_	2.00 mA cm^−2^	0.25 mAh cm^−2^	1000 h	[100]
CNF/MXene@Zn	Zn(OTf)_2_	2 A g^−1^	93.2% after 500 cycles	Zn(OTf)_2_	1.00 mA cm^−2^	\	2800 h	[101]
20F-Zn	ZnSO_4_ + MnSO_4_	2 A g^−1^	68.4% after 500 cycles	ZnSO_4_	2.00 mA cm^−2^	1.00 mAh cm^−2^	1275 h	[102]
PUZ-1@Zn	Zn(OTf)_2_	1 A g^−1^	70.3% after 3400 cycles	Zn(OTf)_2_	0.50 mA cm^−2^	0.50 mAh cm^−2^	1800 h	[103]

## Data Availability

Not applicable.

## References

[1] Blanc L.E., Kundu D., Nazar L.F. (2020). Scientific Challenges for the Implementation of Zn-Ion Batteries. Joule.

[2] Sun H., Huyan Y., Li N., Lei D., Liu H., Hua W., Wei C., Kang F., Wang J.G. (2023). A Seamless Metal-Organic Framework Interphase with Boosted Zn^2+^ Flux and Deposition Kinetics for Long-Living Rechargeable Zn Batteries. Nano Lett..

[3] Zhao R., Yang J., Han X., Wang Y., Ni Q., Hu Z., Wu C., Bai Y. (2023). Stabilizing Zn Metal Anodes via Cation/Anion Regulation toward High Energy Density Zn-Ion Batteries. Adv. Energy Mater..

[4] Jia H., Liu K., Lam Y., Tawiah B., Xin J.H., Nie W., Jiang S.-X. (2022). Fiber-Based Materials for Aqueous Zinc Ion Batteries. Adv. Fiber Mater..

[5] Wang Y., Zhang Y., Cheng H., Ni Z., Wang Y., Xia G., Li X., Zeng X. (2021). Research Progress toward Room Temperature Sodium Sulfur Batteries: A Review. Molecules.

[6] Salgado R.M., Danzi F., Oliveira J.E., El-Azab A., Camanho P.P., Braga M.H. (2021). The Latest Trends in Electric Vehicles Batteries. Molecules.

[7] Chen D., Li Y., Zhang X., Hu S., Yu Y. (2022). Investigation of the discharging behaviors of different doped silicon nanowires in alkaline Si-air batteries. J. Ind. Eng. Chem..

[8] Gao S., Zhao T., Wang D., Huang J., Xiang Y., Yu Y. (2022). Ge nanowires on top of a Ge substrate for applications in anodes of Li and Na ion batteries: A first-principles study. RSC Adv..

[9] Yu Y., Gao S., Hu S. (2021). Si modified by Zn and Fe as anodes in Si-air batteries with ameliorative properties. J. Alloys Compd..

[10] Yu Y., Wang D., Luo J., Xiang Y. (2023). First-principles study of ZIF-8 as anode for Na and K ion batteries. Colloids Surf. A Physicochem. Eng. Asp..

[11] Zhao T., Zhang Y., Wang D., Chen D., Zhang X., Yu Y. (2023). Graphene-coated Ge as anodes in Ge-air batteries with enhanced performance. Carbon.

[12] Yu Y., Hu S. (2021). The applications of semiconductor materials in air batteries. Chin. Chem. Lett..

[13] Liu W., Lu B., Liu X., Gan Y., Zhang S., Shi S. (2021). In Situ Synthesis of the Peapod-Like Cu–SnO_2_@Copper Foam as Anode with Excellent Cycle Stability and High Area Specific Capacity. Adv. Funct. Mater..

[14] Liu W., Chen X., Xiang P., Zhang S., Yan J., Li N., Shi S. (2019). Chemically monodisperse tin nanoparticles on monolithic 3D nanoporous copper for lithium-ion battery anodes with ultralong cycle life and stable lithium storage properties. Nanoscale.

[15] Deng F., Zhang Y., Yu Y. (2023). Conductive Metal–Organic Frameworks for Rechargeable Lithium Batteries. Batteries.

[16] Pan H., Shao Y., Yan P., Cheng Y., Han K., Nie Z., Wang C., Yang J., Li X., Mueller K.T. (2016). Highly Reversible Aqueous Zn/MnO_2_ Energy Storage System from Chemical Conversion Reactions. ECS Meet. Abstr..

[17] Dai Q., Li L., Hoang T.K.A., Tu T., Hu B., Jia Y., Zhang M., Song L., Trudeau M.L. (2022). The secondary aqueous zinc-manganese battery. J. Energy Storage.

[18] Yu X., Liu G., Wang T., Gong H., Qu H., Meng X., He J., Ye J. (2022). Recent Advances in the Research of Photo-Assisted Lithium-Based Rechargeable Batteries. Chem. Asian J..

[19] Lv J., Abbas S.C., Huang Y., Liu Q., Wu M., Wang Y., Dai L. (2018). A photo-responsive bifunctional electrocatalyst for oxygen reduction and evolution reactions. Nano Energy.

[20] Boruah B.D., Mathieson A., Wen B., Feldmann S., Dose W.M., De Volder M. (2020). Photo-rechargeable zinc-ion batteries. Energy Environ. Sci..

[21] Li J., Zhang K., Wang B., Peng H. (2022). Light-Assisted Metal–Air Batteries: Progress, Challenges, and Perspectives. Angew. Chem. Int. Ed..

[22] Song J., Xu K., Liu N., Reed D., Li X. (2021). Crossroads in the renaissance of rechargeable aqueous zinc batteries. Mater. Today.

[23] Huang J., Qiu X., Wang N., Wang Y. (2021). Aqueous rechargeable zinc batteries: Challenges and opportunities. Curr. Opin. Electrochem..

[24] Xu X., Song M., Li M., Xu Y., Sun L., Shi L., Su Y., Lai C., Wang C. (2023). A novel bifunctional zinc gluconate electrolyte for a stable Zn anode. Chem. Eng. J..

[25] Tao F., Liu Y., Ren X., Wang J., Zhou Y., Miao Y., Ren F., Wei S., Ma J. (2022). Different surface modification methods and coating materials of zinc metal anode. J. Energy Chem..

[26] Jian Q., Wan Y., Sun J., Wu M., Zhao T. (2020). A dendrite-free zinc anode for rechargeable aqueous batteries. J. Mater. Chem. A.

[27] Gao X., Li Y., Yin W., Lu X. (2022). Recent Advances of Carbon Materials in Anodes for Aqueous Zinc Ion Batteries. Chem. Rec..

[28] Su Y., Yang X., Zhang Q., Sun J., Liu Z. (2022). Carbon nanomaterials for highly stable Zn anode: Recent progress and future outlook. J. Electroanal. Chem..

[29] Wang Y., Xie J., Luo J., Yu Y., Liu X., Lu X. (2022). Methods for Rational Design of Advanced Zn-Based Batteries. Small Methods.

[30] Li B., Zhang X., Wang T., He Z., Lu B., Liang S., Zhou J. (2021). Interfacial Engineering Strategy for High-Performance Zn Metal Anodes. Nano-Micro Lett..

[31] Shang Y., Kundu D. (2022). Understanding and Performance of the Zinc Anode Cycling in Aqueous Zinc-Ion Batteries and a Roadmap for the Future. Batter. Supercaps.

[32] Liu Y., Liu Y., Wu X. (2022). Toward Long-Life Aqueous Zinc Ion Batteries by Constructing Stable Zinc Anodes. Chem. Rec..

[33] Liang P., Yi J., Liu X., Wu K., Wang Z., Cui J., Liu Y., Wang Y., Xia Y., Zhang J. (2020). Highly Reversible Zn Anode Enabled by Controllable Formation of Nucleation Sites for Zn-Based Batteries. Adv. Funct. Mater..

[34] Tao H., Hou Z., Zhang L., Yang X., Fan L.-Z. (2022). Manipulating alloying reaction to achieve the stable and dendrite-free zinc metal anodes. Chem. Eng. J..

[35] Lu W., Xie C., Zhang H., Li X. (2018). Inhibition of Zinc Dendrite Growth in Zinc-Based Batteries. ChemSusChem.

[36] Li H., Guo C., Zhang T., Xue P., Zhao R., Zhou W., Li W., Elzatahry A., Zhao D., Chao D. (2022). Hierarchical Confinement Effect with Zincophilic and Spatial Traps Stabilized Zn-Based Aqueous Battery. Nano Lett..

[37] Cao P., Zhou H., Zhou X., Du Q., Tang J., Yang J. (2022). Stabilizing Zinc Anodes by a Cotton Towel Separator for Aqueous Zinc-Ion Batteries. ACS Sustain. Chem. Eng..

[38] He Q., Yu B., Li Z., Zhao Y. (2019). Density Functional Theory for Battery Materials. Energy Environ. Mater..

[39] Zhang X., Hu J.P., Fu N., Zhou W.B., Liu B., Deng Q., Wu X.W. (2022). Comprehensive review on zinc-ion battery anode: Challenges and strategies. InfoMat.

[40] Qin R., Wang Y., Yao L., Yang L., Zhao Q., Ding S., Liu L., Pan F. (2022). Progress in interface structure and modification of zinc anode for aqueous batteries. Nano Energy.

[41] Song M., Tan H., Chao D., Fan H.J. (2018). Recent Advances in Zn-Ion Batteries. Adv. Funct. Mater..

[42] Xu C., Li B., Du H., Kang F. (2012). Energetic zinc ion chemistry: The rechargeable zinc ion battery. Angew. Chem. Int. Ed..

[43] Kim E., Choi I., Nam K.W. (2022). Metal–organic framework for dendrite-free anodes in aqueous rechargeable zinc batteries. Electrochim. Acta.

[44] Wang Y., Chen Y., Liu W., Ni X., Qing P., Zhao Q., Wei W., Ji X., Ma J., Chen L. (2021). Uniform and dendrite-free zinc deposition enabled by in situ formed AgZn3 for the zinc metal anode. J. Mater. Chem. A.

[45] Liu H., Wang J.-G., Hua W., Ren L., Sun H., Hou Z., Huyan Y., Cao Y., Wei C., Kang F. (2022). Navigating fast and uniform zinc deposition via a versatile metal–organic complex interphase. Energy Environ. Sci..

[46] Wang J., Yang Y., Zhang Y., Li Y., Sun R., Wang Z., Wang H. (2021). Strategies towards the challenges of zinc metal anode in rechargeable aqueous zinc ion batteries. Energy Storage Mater..

[47] Yuksel R., Buyukcakir O., Seong W.K., Ruoff R.S. (2020). Metal-Organic Framework Integrated Anodes for Aqueous Zinc-Ion Batteries. Adv. Energy Mater..

[48] Zheng J., Zhao Q., Tang T., Yin J., Quilty C.D., Renderos G.D., Liu X., Deng Y., Wang L., Bock D.C. (2019). Reversible epitaxial electrodeposition of metals in battery anodes. Science.

[49] Zheng J., Huang Z., Ming F., Zeng Y., Wei B., Jiang Q., Qi Z., Wang Z., Liang H. (2022). Surface and Interface Engineering of Zn Anodes in Aqueous Rechargeable Zn-Ion Batteries. Small.

[50] Zhao Z., Wang R., Peng C., Chen W., Wu T., Hu B., Weng W., Yao Y., Zeng J., Chen Z. (2021). Horizontally arranged zinc platelet electrodeposits modulated by fluorinated covalent organic framework film for high-rate and durable aqueous zinc ion batteries. Nat. Commun..

[51] Ho V.C., Lim H., Kim M.J., Mun J. (2022). Improving the Performance of Aqueous Zinc-ion Batteries by Inhibiting Zinc Dendrite Growth: Recent Progress. Chem. Asian J..

[52] Pu S.D., Gong C., Tang Y.T., Ning Z., Liu J., Zhang S., Yuan Y., Melvin D., Yang S., Pi L. (2022). Achieving Ultrahigh-Rate Planar and Dendrite-Free Zinc Electroplating for Aqueous Zinc Battery Anodes. Adv. Mater..

[53] Wang N., Wu Z., Long Y., Chen D., Geng C., Liu X., Han D., Zhang J., Tao Y., Yang Q.-H. (2022). MXene-assisted polymer coating from aqueous monomer solution towards dendrite-free zinc anodes. J. Energy Chem..

[54] Li B., Xue J., Han C., Liu N., Ma K., Zhang R., Wu X., Dai L., Wang L., He Z. (2021). A hafnium oxide-coated dendrite-free zinc anode for rechargeable aqueous zinc-ion batteries. J. Colloid Interface Sci..

[55] Zhang H., Li S., Xu L., Momen R., Deng W., Hu J., Zou G., Hou H., Ji X. (2022). High-Yield Carbon Dots Interlayer for Ultra-Stable Zinc Batteries. Adv. Energy Mater..

[56] Zhang S., Ye J., Ao H., Zhang M., Li X., Xu Z., Hou Z., Qian Y. (2022). In-situ formation of hierarchical solid-electrolyte interphase for ultra-long cycling of aqueous zinc-ion batteries. Nano Res..

[57] Shin J., Lee J., Kim Y., Park Y., Kim M., Choi J.W. (2021). Highly Reversible, Grain-Directed Zinc Deposition in Aqueous Zinc Ion Batteries. Adv. Energy Mater..

[58] Luo B., Wang Y., Zheng S., Sun L., Duan G., Lu J., Huang J., Ye Z. (2022). Ion pumping synergy with atomic anchoring for dendrite-free Zn anodes. Energy Storage Mater..

[59] Xue P., Guo C., Li L., Li H., Luo D., Tan L., Chen Z. (2022). A MOF-Derivative Decorated Hierarchical Porous Host Enabling Ultrahigh Rates and Superior Long-Term Cycling of Dendrite-Free Zn Metal Anodes. Adv. Mater..

[60] Wen Q., Fu H., Wang Z.-y., Huang Y.-d., He Z.-j., Yan C., Mao J., Dai K., Zhang X.-h., Zheng J.-C. (2022). A hydrophobic layer of amino acid enabling dendrite-free Zn anodes for aqueous zinc-ion batteries. J. Mater. Chem. A.

[61] Zhao T., Wu H., Wen X., Zhang J., Tang H., Deng Y., Liao S., Tian X. (2022). Recent advances in MOFs/MOF derived nanomaterials toward high-efficiency aqueous zinc ion batteries. Coord. Chem. Rev..

[62] Zenaidee M.A., Loo J.A. (2022). Seeing flying molecular elephants more clearly. Nat. Chem..

[63] Zhao J., Ying Y., Wang G., Hu K., Yuan Y.D., Ye H., Liu Z., Lee J.Y., Zhao D. (2022). Covalent organic framework film protected zinc anode for highly stable rechargeable aqueous zinc-ion batteries. Energy Storage Mater..

[64] Xue R., Kong J., Wu Y., Wang Y., Kong X., Gong M., Zhang L., Lin X., Wang D. (2022). Highly reversible zinc metal anodes enabled by a three-dimensional silver host for aqueous batteries. J. Mater. Chem. A.

[65] Zeng X., Zhao J., Wan Z., Jiang W., Ling M., Yan L., Liang C. (2021). Controllably Electrodepositing ZIF-8 Protective Layer for Highly Reversible Zinc Anode with Ultralong Lifespan. J. Phys. Chem. Lett..

[66] So S., Ahn Y.N., Ko J., Kim I.T., Hur J. (2022). Uniform and oriented zinc deposition induced by artificial Nb2O5 Layer for highly reversible Zn anode in aqueous zinc ion batteries. Energy Storage Mater..

[67] Liu C., Luo Z., Deng W., Wei W., Chen L., Pan A., Ma J., Wang C., Zhu L., Xie L. (2021). Liquid Alloy Interlayer for Aqueous Zinc-Ion Battery. ACS Energy Lett..

[68] Liu B., Wang S., Wang Z., Lei H., Chen Z., Mai W. (2020). Novel 3D Nanoporous Zn-Cu Alloy as Long-Life Anode toward High-Voltage Double Electrolyte Aqueous Zinc-Ion Batteries. Small.

[69] Li B., Yang K., Ma J., Shi P., Chen L., Chen C., Hong X., Cheng X., Tang M.C., He Y.B. (2022). Multicomponent Copper-Zinc Alloy Layer Enabling Ultra-Stable Zinc Metal Anode of Aqueous Zn-ion Battery. Angew. Chem. Int. Ed..

[70] Zhang Y., Yang X., Hu Y., Hu K., Lin X., Liu X., Reddy K.M., Xie G., Qiu H.J. (2022). Highly Strengthened and Toughened Zn-Li-Mn Alloys as Long-Cycling Life and Dendrite-Free Zn Anode for Aqueous Zinc-Ion Batteries. Small.

[71] Zhang L., Zhang B., Zhang T., Li T., Shi T., Li W., Shen T., Huang X., Xu J., Zhang X. (2021). Eliminating Dendrites and Side Reactions via a Multifunctional ZnSe Protective Layer toward Advanced Aqueous Zn Metal Batteries. Adv. Funct. Mater..

[72] Qi Z., Xiong T., Chen T., Yu C., Zhang M., Yang Y., Deng Z., Xiao H., Lee W.S.V., Xue J. (2021). Dendrite-Free Anodes Enabled by a Composite of a ZnAl Alloy with a Copper Mesh for High-Performing Aqueous Zinc-Ion Batteries. ACS Appl. Mater. Interfaces.

[73] Chen Y., Zhao Q., Wang Y., Liu W., Qing P., Chen L. (2021). A dendrite-free Zn@CuxZny composite anode for rechargeable aqueous batteries. Electrochim. Acta.

[74] Tribbia M., Zampardi G., La Mantia F. (2023). Towards the commercialization of rechargeable aqueous zinc ion batteries: The challenge of the zinc electrodeposition at the anode. Curr. Opin. Electrochem..

[75] Liu M., Yao L., Ji Y., Zhang M., Gan Y., Cai Y., Li H., Zhao W., Zhao Y., Zou Z. (2023). Nanoscale Ultrafine Zinc Metal Anodes for High Stability Aqueous Zinc Ion Batteries. Nano Lett..

[76] Zheng S., Zhao W., Chen J., Zhao X., Pan Z., Yang X. (2023). 2D Materials Boost Advanced Zn Anodes: Principles, Advances, and Challenges. Nano-Micro Lett..

[77] Ge J., Zhang Y., Xie Z., Xie H., Chen W., Lu B. (2023). Tailored ZnF2/ZnS-rich interphase for reversible aqueous Zn batteries. Nano Res..

[78] Huang Z., Li H., Yang Z., Wang H., Ding J., Xu L., Tian Y., Mitlin D., Ding J., Hu W. (2022). Nanosecond laser lithography enables concave-convex zinc metal battery anodes with ultrahigh areal capacity. Energy Storage Mater..

[79] Li T.C., Lim Y.V., Xie X., Li X.L., Li G., Fang D., Li Y., Ang Y.S., Ang L.K., Yang H.Y. (2021). ZnSe Modified Zinc Metal Anodes: Toward Enhanced Zincophilicity and Ionic Diffusion. Small.

[80] Meng H., Ran Q., Dai T.Y., Shi H., Zeng S.P., Zhu Y.F., Wen Z., Zhang W., Lang X.Y., Zheng W.T. (2022). Surface-Alloyed Nanoporous Zinc as Reversible and Stable Anodes for High-Performance Aqueous Zinc-Ion Battery. Nano-Micro Lett..

[81] Zhao Q., Liu W., Chen Y., Chen L. (2022). Ultra-stable Zn metal batteries with dendrite-free Cu-Sn alloy induced high-quality composite Zn mesh. Chem. Eng. J..

[82] Wang F., Lu H., Li H., Li J., Wang L., Han D., Gao J., Geng C., Cui C., Zhang Z. (2022). Demonstrating U-shaped zinc deposition with 2D metal-organic framework nanoarrays for dendrite-free zinc batteries. Energy Storage Mater..

[83] Lei L., Chen F., Wu Y., Shen J., Wu X.-J., Wu S., Yuan S. (2022). Surface coatings of two-dimensional metal-organic framework nanosheets enable stable zinc anodes. Sci. China Chem..

[84] He W., Gu T., Xu X., Zuo S., Shen J., Liu J., Zhu M. (2022). Uniform In Situ Grown ZIF-L Layer for Suppressing Hydrogen Evolution and Homogenizing Zn Deposition in Aqueous Zn-Ion Batteries. ACS Appl. Mater. Interfaces.

[85] Tao Y., Zuo S.W., Xiao S.H., Sun P.X., Li N.W., Chen J.S., Zhang H.B., Yu L. (2022). Atomically Dispersed Cu in Zeolitic Imidazolate Framework Nanoflake Array for Dendrite-Free Zn Metal Anode. Small.

[86] Xu Q., Zhou W., Xin T., Zheng Z., Yuan X., Liu J. (2022). Practical Zn anodes enabled by a Ti-MOF-derived coating for aqueous batteries. J. Mater. Chem. A.

[87] Xin T., Wang Y., Xu Q., Shang J., Yuan X., Song W., Liu J. (2022). Forming an Amorphous ZnO Nanosheet Network by Confined Parasitic Reaction for Stabilizing Zn Anodes and Reducing Water Activity. ACS Appl. Energy Mater..

[88] Long Y., Huang X., Li Y., Yi M., Hou J., Zhou X., Hu Q., Zheng Q., Lin D. (2022). In-situ regulation of zinc metal surface for Dendrite-Free Zinc-ion hybrid supercapacitors. J. Colloid Interface Sci..

[89] Deng C., Xie X., Han J., Lu B., Liang S., Zhou J. (2021). Stabilization of Zn Metal Anode through Surface Reconstruction of a Cerium-Based Conversion Film. Adv. Funct. Mater..

[90] Liu C., Li Z., Zhang X., Xu W., Chen W., Zhao K., Wang Y., Hong S., Wu Q., Li M.C. (2022). Synergic Effect of Dendrite-Free and Zinc Gating in Lignin-Containing Cellulose Nanofibers-MXene Layer Enabling Long-Cycle-Life Zinc Metal Batteries. Adv. Sci..

[91] Wang T., Xi Q., Li Y., Fu H., Hua Y., Shankar E.G., Kakarla A.K., Yu J.S. (2022). Regulating Dendrite-Free Zinc Deposition by Red Phosphorous-Derived Artificial Protective Layer for Zinc Metal Batteries. Adv. Sci..

[92] Lu J., Yang J., Zhang Z., Wang C., Xu J., Wang T. (2022). Silk Fibroin Coating Enables Dendrite-free Zinc Anode for Long-Life Aqueous Zinc-Ion Batteries. ChemSusChem.

[93] Qi Z., Xiong T., Yu Z.G., Meng F., Chen B., Xiao H., Xue J. (2023). Suppressing zinc dendrite growth in aqueous battery via Zn–Al alloying with spatially confined zinc reservoirs. J. Power Sources.

[94] Wang Y., Song J., Wong W.Y.R. (2022). Constructing 2D Sandwich-like MOF/MXene Heterostructures for Durable and Fast Aqueous Zinc-Ion Batteries. Angew. Chem. Int. Ed..

[95] Li M., Zhou X., He X., Lai C., Shan B., Wang K., Jiang K. (2023). Controllable CF_4_ Plasma in Situ Modification Strategy Enables Durable Zinc Metal Anode. ACS Appl. Mater. Interfaces.

[96] Yang X., Lv J., Cheng C., Shi Z., Peng J., Chen Z., Lian X., Li W., Zou Y., Zhao Y. (2022). Mosaic Nanocrystalline Graphene Skin Empowers Highly Reversible Zn Metal Anodes. Adv. Sci..

[97] Luan X., Qi L., Zheng Z., Gao Y., Xue Y., Li Y. (2023). Step by Step Induced Growth of Zinc-Metal Interface on Graphdiyne for Aqueous Zinc-Ion Batteries. Angew. Chem. Int. Ed..

[98] Xin W., Xiao J., Li J., Zhang L., Peng H., Yan Z., Zhu Z. (2023). Metal-organic frameworks with carboxyl functionalized channels as multifunctional ion-conductive interphase for highly reversible Zn anode. Energy Storage Mater..

[99] Li J., Zheng Z., Yu Z., She F., Lai L., Prabowo J., Lv W., Wei L., Chen Y. (2023). Stable Zn electrodes enabled by an ultra-thin Zn phosphate protective layer. J. Mater. Chem. A.

[100] Yuan X., Yi J., Li C., Zhao Z., Xiong C. (2023). An artificial β-PVDF nanofiber layer for dendrite-free zinc anode in rechargeable aqueous batteries. J. Mater. Sci..

[101] Xu W., Liao X., Xu W., Zhao K., Yao G., Wu Q. (2023). Ion Selective and water-resistant Cellulose Nanofiber/MXene Membrane Enabled Cycling Zn Anode at High Currents. Adv. Energy Mater..

[102] Wang X., Wang X., Zhou Y., Qi H., Li X., Wei C., Zou T., Wang W., Yang Z. (2023). In-situ construction of multifunctional protection interface for ultra-stable zinc anodes. J. Alloys Compd..

[103] Zhou S., Su Y., Li G., Wang X., Liu D., Zhu G. (2023). Zincophilic polyurethane-based porous film enables dendrite-free zinc anode for reversible aqueous zinc-based batteries. Colloids Surf. A Physicochem. Eng. Asp..

[104] Zhang L., Hou Y. (2021). Comprehensive Analyses of Aqueous Zn Metal Batteries: Characterization Methods, Simulations, and Theoretical Calculations. Adv. Energy Mater..

[105] Yin Y., Wang S., Zhang Q., Song Y., Chang N., Pan Y., Zhang H., Li X. (2020). Dendrite-Free Zinc Deposition Induced by Tin-Modified Multifunctional 3D Host for Stable Zinc-Based Flow Battery. Adv. Mater..

[106] Nie X., Miao L., Yuan W., Ma G., Di S., Wang Y., Shen S., Zhang N. (2022). Cholinium Cations Enable Highly Compact and Dendrite-Free Zn Metal Anodes in Aqueous Electrolytes. Adv. Funct. Mater..

[107] Chen Y., Ma D., Ouyang K., Yang M., Shen S., Wang Y., Mi H., Sun L., He C., Zhang P. (2022). A Multifunctional Anti-Proton Electrolyte for High-Rate and Super-Stable Aqueous Zn-Vanadium Oxide Battery. Nano-Micro Lett..

[108] Yan M., Dong N., Zhao X., Sun Y., Pan H. (2021). Tailoring the Stability and Kinetics of Zn Anodes through Trace Organic Polymer Additives in Dilute Aqueous Electrolyte. ACS Energy Lett..

[109] Qiu M., Sun P., Wang Y., Ma L., Zhi C., Mai W. (2022). Anion-Trap Engineering toward Remarkable Crystallographic Reorientation and Efficient Cation Migration of Zn Ion Batteries. Angew. Chem. Int. Ed..

[110] Zhou L., Wang F., Yang F., Liu X., Yu Y., Zheng D., Lu X. (2022). Unshared Pair Electrons of Zincophilic Lewis Base Enable Long-life Zn Anodes under “Three High” Conditions. Angew. Chem. Int. Ed..

[111] Zhao M., Rong J., Huo F., Lv Y., Yue B., Xiao Y., Chen Y., Hou G., Qiu J., Chen S. (2022). Semi-Immobilized Ionic Liquid Regulator with Fast Kinetics toward Highly Stable Zinc Anode under −35 to 60 °C. Adv. Mater..

[112] Yao N., Chen X., Fu Z.H., Zhang Q. (2022). Applying Classical, Ab Initio, and Machine-Learning Molecular Dynamics Simulations to the Liquid Electrolyte for Rechargeable Batteries. Chem. Rev..

